# Gastrointestinal symptoms among endometriosis patients—A case-cohort study

**DOI:** 10.1186/s12905-015-0213-2

**Published:** 2015-08-13

**Authors:** Malin Ek, Bodil Roth, Per Ekström, Lil Valentin, Mariette Bengtsson, Bodil Ohlsson

**Affiliations:** Department of Clinical Sciences, Division of Internal Medicine, Skåne University Hospital, Lund University, 205 02, Malmö, Sweden; Department of Clinical Sciences, Division of Gynecology, Skåne University Hospital, Lund University, 205 02 Malmö, Sweden; Faculty of Health and Society, Institution of Care Science, Malmö University, Malmö, Sweden

**Keywords:** Abdominal pain, Endometriosis, Gastrointestinal symptoms, Gonadotropin-releasing hormone, Menstruation, Opioids

## Abstract

**Background:**

Women with endometriosis often experience gastrointestinal symptoms. Gonadotropin-releasing hormone (GnRH) analogs are used to treat endometriosis; however, some patients develop gastrointestinal dysmotility following this treatment. The aims of the present study were to investigate gastrointestinal symptoms among patients with endometriosis and to examine whether symptoms were associated with menstruation, localization of endometriosis lesions, or treatment with either opioids or GnRH analogs, and if hormonal treatment affected the symptoms.

**Methods:**

All patients with diagnosed endometriosis at the Department of Gynecology were invited to participate in the study. Gastrointestinal symptoms were registered using the Visual Analogue Scale for Irritable Bowel Syndrome (VAS-IBS); socioeconomic and medical histories were compiled using a clinical data survey. Data were compared to a control group from the general population.

**Results:**

A total of 109 patients and 65 controls were investigated. Compared to controls, patients with endometriosis experienced significantly aggravated abdominal pain (*P* = 0.001), constipation (*P* = 0.009), bloating and flatulence (*P* = 0.000), defecation urgency (*P* = 0.010), and sensation of incomplete evacuation (*P* = 0.050), with impaired psychological well-being (*P* = 0.005) and greater intestinal symptom influence on their daily lives (*P* = 0.001). The symptoms were not associated with menstruation or localization of endometriosis lesions, except increased nausea and vomiting (*P* = 0.010) in patients with bowel-associated lesions. Half of the patients were able to differentiate between abdominal pain from endometriosis and from the gastrointestinal tract. Patients using opioids experienced more severe symptoms than patients not using opioids, and patients with current or previous use of GnRH analogs had more severe abdominal pain than the other patients (*P* = 0.024). Initiation of either combined oral contraceptives or progesterone for endometriosis had no effect on gastrointestinal symptoms when the patients were followed prospectively.

**Conclusions:**

The majority of endometriosis patients experience more severe gastrointestinal symptoms than controls. A poor association between symptoms and lesion localization was found, indicating existing comorbidity between endometriosis and irritable bowel syndrome (IBS). Treatment with opioids or GnRH analogs is associated with aggravated gastrointestinal symptoms.

## Background

Endometriosis is a benign, gynecological disease associated with the primary symptoms of chronic pelvic pain, deep dyspareunia, and dysmenorrhea [1]. The prevalence of endometriosis differs in the literature, but is estimated to affect approximately 7–10 % of women [2]. Clinically, women with endometriosis commonly experience gastrointestinal symptoms, and one study has shown that gastrointestinal symptoms are almost as common as gynecological symptoms in these patients [3].

Gastrointestinal symptoms among patients with endometriosis described in the literature include abdominal pain, bloating, nausea, constipation, vomiting, painful bowel movements, and diarrhea [3–5]. However, reported symptoms differ between studies. Aggravated symptoms during menstruation have been reported [4, 6, 7] such as cyclic-related bloating and constipation [4]. Fauconnier et al. [7] concluded that symptoms including diarrhea, constipation, and colic rectal pain were more frequent among patients with endometriosis lesions within or close to the bowel. In contrast, Maroun et al. [3] reported gastrointestinal symptoms to be primarily independent of localization of endometriosis lesions in relation to the bowel. Different explanations concerning the occurrence of these symptoms include: endometriosis lesions cause inflammatory activity and local prostaglandin release, which can alter bowel function [8]; endometriosis lesions within the bowel cause symptoms due to mechanical obstruction or cyclic micro-hemorrhages [9]; or there is an existing comorbidity between endometriosis and irritable bowel syndrome (IBS) [8].

Gonadotropin-releasing hormone (GnRH) is a hypothalamic hormone [10], which has also been shown to be present in neurons in the human enteric nervous system (ENS) [11]. Recent studies have suggested a link between GnRH and gastrointestinal function [12, 13], and some patients develop severe dysmotility in the form of chronic intestinal pseudo-obstruction (CIPO) or enteric dysmotility (ED) after treatment with GnRH analogs in relation to in vitro fertilization (IVF) or endometriosis [14]. Full-thickness biopsies of the bowel wall have shown enteric neurodegeneration with almost total absence of GnRH-containing neurons [11, 14, 15]. Hammar et al. [13] investigated 124 patients before and after treatment with GnRH analogs and concluded that there was a significant exacerbation of gastrointestinal symptoms after treatment, and abdominal pain was still exacerbated at 5-year follow-up.

The aim of the present study was to investigate the severity of gastrointestinal symptoms among patients with endometriosis compared to a control group from the general population. Furthermore, an association between symptoms and menstruation, localization of endometriosis lesions, and treatment with opioids or GnRH analogs was investigated. The final aim was to investigate women initiating hormonal treatment to examine whether this treatment had an impact on gastrointestinal symptoms over time.

## Methods

This study was approved by the Ethics Review Board of Lund University, Dnr 2012/564, and performed in accordance with the declaration of Helsinki. All subjects gave their written, informed consent before inclusion in the study.

### Patients

Patients who had sought treatment for endometriosis in the past 5 years were recruited from the Department of Gynecology at Skåne University Hospital in Malmö. The patients were identified with the International Statistical Classification of Diseases and Related Health Problems, ICD-10, N80.1, 80.4, 80.5, 80.8, and 80.9 from Skåne University Hospital medical records. The clinic’s catchment area is the southernmost districts of Sweden. The recruitment was conducted continuously from March 2013 through July 2014. Inclusion criteria were a diagnosis of endometriosis made by laparoscopy, ultrasonography, or a definite clinical diagnosis made by a gynecologist. The included patients were also required to comprehend the Swedish or English language. Exclusion criteria were an uncertain diagnosis of endometriosis, patients living too far from the geographical area of Skåne University Hospital, multi-morbidity, current pregnancy, or a diagnosis of cancer, inflammatory bowel disease, multiple sclerosis, psychiatric disease, or rheumatoid arthritis.

### Controls

Data regarding control subjects was obtained from a previous study conducted by Hammar et al. [13]. In this study, control subjects were randomly acquired from the Swedish Population Registry, and 248 subjects were contacted. In total, after one reminder, 29 questionnaires were returned. Because of the low response rate, further controls were recruited amongst female hospital staff. In total, 65 women representing the general population, with a mean age of 40 ± 9 years, were recruited and completed the Visual Analogue Scale for Irritable Bowel Syndrome (VAS-IBS).

### Study design

The patients were contacted via mail, and within a week, they were also contacted via telephone. After agreement to participate in the study, an appointment for an interview and a blood draw was done 1–4 weeks after inclusion in the study. The questionnaire, Visual Analogue Scale for Irritable Bowel Syndrome (VAS-IBS), and a clinical data survey were sent via mail, with instructions to complete these questionnaires at a maximum of 1 week before the appointment. At the hospital visit, all patients were interviewed regarding previous treatment for endometriosis and their position in the menstrual cycle at the time of completing the VAS-IBS. The patients who were included at the first contact with the Department of Gynecology also completed the VAS-IBS questionnaire and the clinical data survey, were interviewed and had blood samples drawn at 3 and 6 months after their first visit.

A review of the patients’ medical journals was conducted to investigate the localization of endometriosis lesions, current hormonal treatment, and whether they had undergone diagnostic or operative laparoscopy. A lesion was confirmed when it was seen macroscopically during laparoscopy, visualized by ultrasonography, or in a few cases by palpation conducted by a gynecologist.

### Questionnaires

#### Clinical data survey

The clinical data survey addresses socioeconomic factors, physical exercise, nicotine- and alcohol habits, current diseases, and medication, as well as questions about gastrointestinal symptoms such as onset and triggers, whether the subject can differentiate between the abdominal pain from endometriosis and the gastrointestinal tract, and whether pharmacological treatment was used because of the complaints.

Three questions were added after the study was initiated: which year endometriosis-associated symptoms began; which year gastrointestinal symptoms began; and whether the patient could differentiate symptoms from endometriosis and symptoms from the gastrointestinal tract. Therefore, an additional survey with these questions was sent by mail to the 21 first participants already included in the study.

#### Visual Analogue Scale for Irritable Bowel Syndrome

The VAS-IBS was used to investigate gastrointestinal complaints in the study groups. It is a validated questionnaire for estimation of the most common gastrointestinal complaints in patients with non-organic, functional bowel disease [16]. This scale has also been validated for estimation of symptoms over time [17]. The seven items measured in the VAS-IBS address the symptoms abdominal pain, diarrhea, constipation, bloating and flatulence, nausea and vomiting, psychological well-being, and intestinal symptoms’ influence on daily life. These items were measured on a scale from 0 to 100, where 0 represents severe problems and 100 represents a complete lack of problems. An additional two questions, if the subject experienced defecation urgency and had a sensation of incomplete evacuation, were answered with yes or no.

### Statistical methods

The data was analyzed using the statistical software package SPSS for Windows (release 22.0; IBM). Because the controls were slightly older than the endometriosis patients, variables were age-standardized using a linear regression model into which age was added as a covariate and the variables were then expressed as z-scores. When comparing VAS scores between groups, the age-standardized values were used. All variables were analyzed for normal distribution using the Kolomogorov-Smirnov test. As normality was rejected, except for age, the Mann-Whitney *U*-test and the Fisher’s exact test were used to compare different groups. Student *t*-test was used to perform a failure analysis. To calculate differences within the group, Friedman’s test and Wilcoxon’s signed rank test were used. Spearman’s correlation test was used for correlations between age and symptoms. Values were expressed as mean ± standard deviation (SD), median [interquartile range (IQR)], or number (n) and percent (%). *P* ≤ 0.05 was considered statistically significant.

## Results

### Patient characteristics

A total of 627 patients with suspected endometriosis were identified. Of these, 320 were excluded because they either did not fulfill the inclusion criteria or they fulfilled the exclusion criteria. The most common reason for exclusion was uncertainty about the diagnosis. Then, 307 patients remained who fulfilled the inclusion criteria. Of these, 198 patients were excluded since 144 were not willing to participate, 49 had moved from the region, and 4 denied the diagnosis when contacted. Thus, 109 could finally be included in the study. Eighteen of these patients were followed prospectively regarding their gastrointestinal symptoms, and 14 of these initiated a new hormonal treatment for endometriosis during the study period.

The mean age of the 109 included patients was 37 ± 7 years. The median duration of endometriosis was 8.5 (4.0–16.0) years. Patients who were unwilling to participate in the study were younger (35 ± 6 years) than those who participated (*P* = 0.014). Almost two-thirds of the patients had a degree from a university or a college. The vast majority had never smoked and consumed less than one standard glass of alcohol a week (Table 1). Aside from endometriosis the majority of patients were healthy (*n* = 57), and the most common diseases reported were allergies or bronchial asthma (*n* = 18) followed by migraine (*n* = 7) and irritable bowel syndrome (IBS) (*n* = 6).

**Table 1:** Patient characteristics

	Patients with endometriosis *n* = 109
Age (years)	36.78 ± 7.39
BMI (kg/m^2^), missing value = 4	24.00 (22.00–26.00)
Marital status (n, %), missing value = 1	
Living alone	36, 33.0
Married or living with a partner	70, 64.2
Living with parents	2, 1.8
Education (n, %)	
Not completed elementary school	1, 0.9
Completed elementary school	2, 1.8
Completed secondary school	20, 18.3
More than a year of further education after secondary school	24, 22.0
Degree from university or college	62, 56.9
Occupation (n, %), missing value = 1	
Work full time	62, 56.9
Work 99–51 %	22, 20.2
Work 1–50 %	8, 7.3
On sick-leave	5, 4.6
Retired	1, 0.9
Unemployed	1, 0.9
Student	9, 8.3
Smoking habits (n, %)	
Smoke regularly	8, 7.3
Smoke occasionally	10, 9.2
Do not smoke, but have been a smoker	21, 19.3
Have never smoked	70, 64.2
Snuff habits (n, %)	
Snuff regularly	6, 5.5
Do not snuff but have been a regular snuff user	3, 2.8
Have never used snuff	100, 91.7
Alcohol use (n, %)	
Less than one standard glass a week	68, 62.4
1–4 standard glasses a week	31, 28.4
5–9 standard glasses a week	10, 9.2
Physical exercise (n, %)	
No time	18, 16.5
Less than 30 minutes a week	16, 14.7
30–90 minutes a week	37, 33.9
More than 90 minutes a week	38, 34.9

*BMI* = body mass index; *n, %* = number and percentage of patients. One standard glass of alcohol contains 12 grams of pure alcohol. Physical exercise was defined as physical activity that results in shortness of breath. Values are given as mean ± standard deviation, median [interquartile range (IQR)] and number, %

Almost all patients had received hormonal treatment for endometriosis, most commonly combined oral contraceptives (Table 2). Of the 109 patients, 87 had laparoscopically-verified endometriosis, 21 had received their diagnosis by ultrasonography, and only one was diagnosed clinically. The most common localization of endometriosis lesions was in the ovaries (*n* = 83), followed by the peritoneum (*n* = 21), the bowel (*n* = 18), and the Pouch of Douglas (*n* = 16).

**Table 2:** Hormonal treatment of endometriosis

	Patients with endometriosis *n* = 109
Previous or current hormonal treatment (n)	
Any type of hormonal treatment	106
Combined oral contraceptives	90
GnRH analogs	55
Progesterone	55
Estrogen	2
Current hormonal treatment (n), missing value = 23	
Combined oral contraceptives	27
Progesterone	24
No hormonal treatment	24
GnRH analogs	9
Estrogen	2
Onset of gastrointestinal symptoms after hormonal treatment (Yes/No/Unknown)	23/27/59
Type of hormonal treatment causing symptoms (n)	
After/during IVF	6
After cessation of combined oral contraceptives	5
After progesterone medication	3
After or during usage of an IUD	2
After GnRH treatment	2
After Pergotime (Klomifen) treatment	1

*IVF* = in vitro fertilization; *GnRH* = gonadotropin-releasing hormone; *IUD* = intrauterine device; *n* = number of patients. Patients presented can have had more than one hormonal treatment and have stated more than one hormonal treatment as the onset trigger for gastrointestinal symptoms. Patients assigned to the group “GnRH analogs” are those who reported GnRH analogs when asked about hormonal treatment and those who reported having undergone IVF. Values are given as number

### Patients’ gastrointestinal complaints

Of the patients, 85 % reported gastrointestinal complaints during the past year. The onset of symptoms had been gradual for a majority. The median duration of gastrointestinal symptoms was 5 months shorter than the median duration of endometriosis. One-third of the patients’ complaints had been diagnosed as IBS or endometriosis, but one out of five reported never having received any diagnosis for the symptoms. However, almost half of the patients reported having the ability to differentiate between pain symptoms from endometriosis and from the gastrointestinal tract. A variety of different triggers for the symptoms were described (Table 3). Nearly half of the patients had received pharmacological treatment for their gastrointestinal complaints; slightly less than one out of five had been treated with opioids, and almost half of the patients had been treated with other drugs than opioids, for example nonsteroidal anti-inflammatory drugs (NSAID), paracetamol, or anti-depressant medication. However, in many cases, it was not obvious whether the anti-depressant drugs were given for treatment of abdominal pain or depression (Table 4).

**Table 3:** Characterization of gastrointestinal symptoms

	Patients with endometriosis *n* = 109
Onset of gastrointestinal symptoms (n, %), missing value = 19	
Gradual	70, 64.2
Rapid	20, 18.3
Duration of gastrointestinal complaints (years)	8.00 (4.00–15.50)
The evolution of symptoms over time (n, %), missing value = 17	
Constant complaints	14, 12.8
Intermittent complaints	45, 41.3
Progressively worse	33, 30.3
Gastrointestinal complaints during the last year (Yes/No)	93/16
Ability to differentiate between pain due to endometriosis and from the gastrointestinal tract (Yes/No/Unknown)	53/32/24
Has changed occupation because of complaints (Yes/No/Unknown)	5/101/3
Relatives with similar complaints (Yes/No/Unknown)	46/47/16
Experience a trigger for the complaints (Yes/No/Unknown)	35/55/19
Triggers for gastrointestinal complaints (n)	
Menstruation	13
Stress	6
Abdominal surgery	4
Cessation of combined oral contraceptives	4
Endometriosis	3
Side-effects from medications	2
Food	1
Sexual activity	1
Pregnancy	1
Gastrointestinal infection	1
Diagnosis (n)	
Have not been given any name	21
Endometriosis	16
IBS	16
Gastritis	6
Sensitive stomach/bowels	3
Chronic constipation	2
Reflux, dyspepsia	2
Stress	1
Allergy	1
Bowel spasm	1
Stricture	1

*IBS* = irritable bowel syndrome, *n*, % = number and percentage of subjects. Patients presented can have more than one trigger for gastrointestinal symptoms and more than one diagnosis ascribed to the symptom. Values are given as median [interquartile range (IQR)] and number, %

**Table 4:** Examinations, medical treatment, and surgery due to gastrointestinal symptoms

	Patients with endometriosis*n* = 109
Examinations conducted because of gastrointestinal symptoms (n)	
Diagnostic laparoscopy (without surgery)	30
No examinations conducted	23
Ultrasonography	17
Colonoscopy	13
Gastroscopy	12
Unspecified examinations (including x-ray and endoscopy)	9
Rectoscopy	8
Previous or current pharmacological treatment because of gastrointestinal complaints (Yes/No/Unknown)	
Opioids	20/88/1
Other analgesic treatment than opioids	21/78/10
Other pharmacological treatment than analgesics	29/70/10
Types of pharmacological treatment other than opioids for gastrointestinal complaints (n)	
NSAIDs	16
Bulking agents or laxatives	11
PPIs	10
Paracetamol	9
Loperamide	2
Gabapentin or pregabalin	2
Natural remedies or probiotics	2
Cyanocobalamin	1
Diazepam	1
Papaverine	1
Types of current medication (n)	
Combined oral contraceptives	23
NSAIDs	20
Antidepressants (including SSRIs, venlafaxin, and mirtazapin)	19
Opioids	14
Paracetamol	14
Allergy and asthma medication	11
Have undergone abdominal surgery (Yes/No)	98/11
Type of abdominal surgery (n)	
Laproscopic surgery (because of endometriosis)	71
Unspecified	43
Sectio	11
Appendectomy	8
Hysterectomy	8
Removal of ovaries and/or oviducts	6

*NSAID* = non-steroidal anti-inflammatory drugs; *SSRIs* = selective serotonin re-uptake inhibitors; *PPIs* = proton pump inhibitors; *GnRH* = gonadotropin releasing hormone. The six most common currently used medications and abdominal surgery are reported. Patients could have undergone more than one examination, used more than one medication, and had more than one type of abdominal surgery conducted. Values are given as number

### Gastrointestinal symptoms measured by the Visual Analogue Scale for Irritable Bowel Syndrome

Patients with endometriosis experienced aggravated symptoms compared to controls regarding abdominal pain, constipation, bloating and flatulence, and impaired psychological well-being and greater influence of intestinal symptoms on daily life. The endometriosis patients also experienced defecation urgency and a sensation of incomplete evacuation significantly more often (Table 5). Increasing patient age correlated with less diarrhea (*rs* = 0.281, *P* = 0.03), less bloating and flatulence (*rs* = 0.239, *P* = 0.013), less nausea and vomiting (*rs* = 0.286, *P* = 0.003), and better psychological well-being (*rs* = 0.295, *P* = 0.002), whereas age in controls did not correlate with symptoms (data not shown).

**Table 5:** Gastrointestinal symptoms measured by the Visual Analogue Scale for Irritable Bowel Syndrome

	Patients with endometriosis *n* = 109	Controls *n* = 65	*P* value
Age (years)	36.78 ± 7.39	40.20 ± 8.75	0.036
Abdominal pain			
Z-score	−0.68 (−2.12–0.64)	0.30 (−0.43–0.70)	0.001
Absolute score	65.00 (30.00–100.00)	93.00 (73.00–98.00)	
Missing value	2	8	
Diarrhea			
Z-score	0.25 (−1.18–0.69)	0.44 (−0.02–0.65)	0.617
Absolute score	87.00 (45.00–100.00)	95.00 (80.00–98.00)	
Missing value	2	10	
Constipation			
Z-score	−0.29 (−2.11–0.57)	0.43 (0.03–0.58)	0.009
Absolute score	80.00 (30.00–100.00)	95.00 (83.00–98.00)	
Missing value	2	8	
Bloating and flatulence			
Z-score	−0.94 (−1.8–0.46)	0.41 (−1.08–0.85)	0.000
Absolute score	45.00 (20.00–85.00)	81.50 (41.75–96.00)	
Missing value	2	7	
Nausea and vomiting			
Z-score	0.33 (−0.95–0.50)	0.37 (0.11–0.48)	0.284
Absolute score	95.00 (65.00–100.00)	98.00 (92.00–99.00)	
Missing value	2	10	
Psychological well-being			
Z-score	−0.52 (−2.54–0.91)	0.19 (−0.57–0.86)	0.005
Absolute score	75.00 (40.00–95.00)	85.00 (74.00–96.00)	
Missing value	2	4	
Influence on daily life			
Z-score	−0.42 (−1.83–0.57)	0.35 (−0.27–0.67)	0.001
Absolute score	70.00 (30.00–100.00)	96.00 (75.00–98.00)	
Missing value	2	7	
Defecation urgency			
(Yes/No/Unknown)	37/66/6	11/52/2	0.010
Sensation of incomplete evacuation			
(Yes/No/Unknown)	53/50/6	17/46/2	0.050

*Z-score* = standard score. The z-scores were used for calculations with the Mann–Whitney *U*-test. Fisher’s exact test was used to calculate dichotomous variables. *P* ≤ 0.05 was considered statistically significant. Values are given as mean ± standard deviation, median [interquartile range (IQR)], and number

The patients with lesions within the bowel, or in close proximity to the bowel (the pouch of Douglas, the posterior wall of the vagina, or the recto-vaginal septum) (*n* = 34), scored more severe symptoms regarding nausea and vomiting than the other patients [69.71 (33.75–100.00) vs. 84.36 (75.00–100.00); *P* = 0.010]. None of the other symptoms differed depending on localization of the lesions (data not shown).

The patients currently using opioids scored more severe symptoms than the other patients regarding all symptoms except sensation of incomplete evacuation (Table 6). The patients who were not currently using opioids scored more severe symptoms than controls regarding abdominal pain [66.12 (45.00–100.00) vs. 81.40 (73.00–98.00); *P* = 0.013], bloating and flatulence [53.96 (20.00–90.00) vs. 71.72 (41.75–96.00); *P* = 0.005], intestinal symptoms’ influence on daily life [66.01 (35.00–100.00) vs. 81.28 (75.00–98.00); *P* = 0.011], and sensation of incomplete evacuation [45 (47.4 %) vs. 17 (26.2 %); *P* = 0.016]. The following parameters all showed tendency towards significant differences between patients not using opioids and controls: constipation [68.88 (45.00–100.00) vs. 84.47 (83.00–98.00); *P* = 0.051], psychological well-being [68.74 (50.00–100.00) vs. 82.07 (74.00–96.00); *P* = 0.064], and defecation urgency [29 (30.5 %) vs. 11 (16.9 %); *P* = 0.066].

**Table 6:** Gastrointestinal complaints among patients with endometriosis in relation to opioid use

	Patients with current opioid use *n* = 13	Patients with no current opioid use *n* = 94	*P* value
Abdominal pain			
Absolute score	30.00 (10.00–57.50)	70.00 (45.00–100.00)	0.002
Diarrhea			
Absolute score	75.00 (19.00–93.50)	90.00 (60.00–100.00)	0.046
Constipation			
Absolute score	25.00 (10.00–48.00)	80.00 (45.00–100.00)	0.002
Bloating and flatulence			
Absolute score	25.00 (15.50–40.00)	50.00 (20.00–90.00)	0.012
Nausea and vomiting			
Absolute score	50.00 (20.00–65.00)	100.00 (75.00–100.00)	0.000
Psychological well-being			
Absolute score	30.00 (5.00–57.50)	75.00 (50.00–100.00)	0.000
Influence on daily life			
Absolute score	25.00 (7.50–60.00)	75.00 (35.00–100.00)	0.002
Defecation urgency (Yes/No/Unknown)	8/4/2	29/62/4	0.015
Sensation of incomplete evacuation (Yes/No)	8/4/2	45/46/4	0.145

Calculations were made using the Mann Whitney *U*-test. Fisher’s exact test was used to calculate dichotomous variables. *P* ≤ 0.05 was considered statistically significant. Values are given as median [interquartile range (IQR)] and number

The patients who currently received hormonal treatment for endometriosis (combined oral contraceptives, progesterone, estrogen, or GnRH analogs) (*n* = 62) did not experience more severe gastrointestinal symptoms than the other patients (*n* = 24) (data not shown). However, when comparing patients with current or previous GnRH treatment (*n* = 55) towards the other patients (*n* = 51), there was a higher degree of abdominal pain among the first group [55.35 (25.00–91.25) vs. 69.51 (50.00–100.00); *P* = 0.024]. Among those who had no hormonal treatment, the patients who were currently menstruating (*n* = 3), were compared to those who did not menstruate (*n* = 21), and no significant differences regarding symptoms between the groups were found (data not shown).

### Prospective follow-up of gastrointestinal symptoms during treatment

Of the 14 patients initiating a new hormonal treatment for endometriosis, the only significant effect was improved psychological well-being from 0 to 3 months, followed by impaired well-being from 3 to 6 months (Friedman’s test; *P* = 0.006). When performing subgroup analyses, the patients who had initiated a new treatment with combined oral contraceptives (*n* = 5) did not experience any significant reduction in any of the symptoms over time (data not shown). Subgroup analyses in patients who had initiated treatment with progesterone (*n* = 6) showed a significant effect regarding psychological well-being, which had improved at the 3-month follow-up but was impaired at the 6-month follow-up (Friedman’s test; *P* = 0.016).

## Discussion

Compared to controls, endometriosis patients experience more abdominal pain, constipation, bloating and flatulence, influence of intestinal symptoms on daily life, defecation urgency, sensation of incomplete evacuation, and decreased psychological well-being. Localization of endometriosis lesions showed no association with symptoms, except lesions within or close to the bowel, which were associated with more nausea and vomiting. Patients using opioids had more severe symptoms than patients not using opioids, and patients treated with GnRH analogs had more abdominal pain than the other patients. A prospective analysis of patients initiating a new hormonal treatment of endometriosis during the study showed no impact from treatment of gastrointestinal symptoms over time. In contrast, prior studies have shown that progestins relief gastrointestinal symptoms related to the menstrual cycle, as well as overall diarrhea and intestinal cramping [18]. However, the latter study could not detect any effect of progestins on overall constipation, abdominal bloating and feeling of incomplete evacuation [18]. The difference between studies is that the present patients did not experience diarrhea. Thus, hormonal treatment may have its main effect on endometriosis and endometriosis-related symptoms, and less effect on overall gastrointestinal symptoms.

To the best of our knowledge, this is the first study that has investigated gastrointestinal symptoms among patients with endometriosis using a continuous scale, the VAS-IBS. Also, to the best of our knowledge, no study has investigated the symptoms in respect to use of opioids and GnRH analogs.

The findings of the present study partially support the findings of previous studies in regard to bloating, abdominal pain, and constipation among endometriosis patients [3, 5]. However, in other studies, diarrhea, nausea, and vomiting were reported to be common [3, 4]; however, these symptoms were not more frequent among patients than controls in the current study. Previous studies have reported exacerbation of symptoms during menstruation [4, 6, 7]. This phenomenon does not differentiate between endometriosis and IBS, since not only abdominal pain due to endometriosis, but also gastrointestinal symptoms due to functional bowel diseases have been shown to increase during menstruation [19]. Symptoms were not found to be more severe during menstruation in this study, which can depend on few menstruating (*n* = 3) vs. non-menstruating (*n* = 21) patients in the group of non-hormonal treatment, since some patients experienced menstruation as a trigger factor for gastrointestinal symptoms.

Symptoms including diarrhea, constipation, and colic rectal pain have been reported to be more common among patients with endometriosis lesions within or close to the bowel than among patients with distant lesions [7, 9]. However, other studies have not found this association [3]. In the current study, nausea and vomiting were associated with bowel-related lesions, but no other symptom association was detected. These findings, and the fact that hormonal endometriosis treatment did not influence gastrointestinal symptoms over time, indicate an existing comorbidity between endometriosis and IBS.

One explanation for this possible comorbidity is an immunological link, involving increased mast cell activation. In IBS, colonic mast cell activation and mediator release close to mucosal innervation have been reported [20], and in deep infiltrating endometriosis, the presence of an increased number of activated mast cells close to nerves have been described [21]. Issa et al. [22] suggested that the visceral hypersensitivity found in both patients with IBS and endometriosis could amplify gastrointestinal symptoms in patients with endometriosis. A hormonal link is also plausible; GnRH-containing neurons [11] and receptors for LH [23] have been shown to be present in the human gastrointestinal tract as well as in the pelvic organs [24, 25]. IBS is predominantly diagnosed in women, with a female to male ratio ranging from 2:1 to 4:1, depending on the diagnostic procedure [26]. Female sex hormones have been suggested to be involved in IBS-associated pain, since fluctuation in IBS symptoms has been reported with exacerbation of abdominal pain during menstruation [19]. However, the mechanisms underlying these findings are unclear [26]. Exacerbation of gastrointestinal symptoms in endometriosis patients during menstruation has also been reported [4, 6, 7]. Another explanation for the symptoms could be a yet undefined gastrointestinal disease or undiscovered endometriosis in the intestinal wall.

Use of opioids is known to cause gastrointestinal symptoms such as constipation, nausea, and abdominal pain, in severe cases referred to as narcotic bowel syndrome [27]. Almost 20 % of the patients had been prescribed opioids because of abdominal pain, and patients with current opioid use had more severe symptoms on almost all parameters measured by the VAS-IBS than patients not using opioids. One explanation to these findings is that the patients with severe symptoms were prescribed opioids, but another explanation is that opioids could cause or aggravate symptoms. A conclusion to be drawn is that many endometriosis patients may not benefit from opioid treatment. Perhaps, opioids have no or weak positive impact on gastrointestinal symptoms; they possibly may even exacerbate them. Thus, all initiating of opioid treatment in this patient group must be carefully evaluated before continued. When excluding current opioid-users, the endometriosis patients still had more severe symptoms than controls, even though the *P* values were slightly higher than 0.05 in some parameters.

GnRH has been shown to be present in the ENS [11] and has been linked to gastrointestinal function [12, 13], but its role in gut physiology and pathophysiology has not been completely elucidated. Hammar et al. [13] reported increased symptoms of constipation, as well as nausea and vomiting among women after treatment with GnRH analogs, with exacerbation of abdominal pain showing a tendency towards significance. At 5-year follow-up, the patients had more abdominal pain than they experienced prior to GnRH treatment [13], and some patients have developed severe dysmotility secondary to repeated or prolonged GnRH treatment [11, 14, 15]. In the present study, patients treated with GnRH analogs had more abdominal pain than the other patients. This could have several explanations; one is that patients with more severe symptoms received GnRH treatment, but another plausible explanation is that the symptoms are due to side effects evoked by GnRH on the ENS. Further research on GnRH and its role in gastrointestinal function and dysfunction is needed.

The current study has several limitations. Approximately half of the patients identified as fulfilling inclusion criteria declined to participate. One can hypothesize that the patients who agreed to participate experienced more severe symptoms, making them more prone to report them. Another weakness is that the patient group and control group were not matched regarding age. To minimize the impact of this factor, z-scores were calculated and analyzed. However, age only correlated with symptoms in patients and not in controls, and the patients who declined to participate were slightly younger, which correlated with more severe symptoms. The patients reported pharmacological treatment of endometriosis and gastrointestinal symptoms, and as the majority of patients most likely had no medical education, one can assume that they less accurately reported their symptoms. To minimize inaccuracy, the pharmacological treatment was discussed at the interview. When asked to measure abdominal pain on the VAS-IBS, it is not clear whether patients responded regarding pain related to endometriosis or gastrointestinal symptoms. However, almost half of the patients reported having the ability to differentiate pain from endometriosis from that of the gastrointestinal tract. The patients appear to be a selected group with high education and a healthy lifestyle; they reported low consumption of alcohol and tobacco. However, these habits could also be due to the fact that alcohol and smoking exacerbate their symptoms.

This study has several clinical impacts. Endometriosis is a disease with a considerable diagnostic delay [28], and an extended knowledge of symptoms associated with the disease could potentially contribute to reducing this delay. It is also of importance to acknowledge these symptoms in order to provide adequate pharmacological treatment, especially since hormonal treatment of endometriosis appeared to have no effect on gastrointestinal symptoms. The findings also indicate the importance of conservativeness in prescribing opioids to this group of patients, especially if the indication is gastrointestinal symptoms, because opioids appear to have no or weak effect on these symptoms. Physicians must carefully evaluate the effect of opioids and should withdraw opioid treatment when the effect is uncertain.

## Conclusions

A large proportion of patients with endometriosis suffer from gastrointestinal symptoms. Compared to controls, patients with endometriosis suffer from more severe abdominal pain, constipation, bloating and flatulence, impaired psychological well-being, influence of symptoms on daily life, defecation urgency, and sensation of incomplete evacuation. The location of the endometriosis lesions is not associated with symptoms, except increased nausea and vomiting among patients with lesions within or close to the bowel. Patients treated with opioids have more severe symptoms than patients not treated with opioids, and current or previous treatment with GnRH analogs is associated with increased abdominal pain. Initiating a new hormonal treatment of endometriosis has no impact on gastrointestinal symptoms over time.

## Abbreviations

CIPO: Chronic intestinal pseudo-obstruction
GnRH: Gonadotropin-releasing hormone
IVF: In vitro fertilization
LH: Luteinizing hormone
VAS-IBS: Visual analogue scale for irritable bowel syndrome
